# Supplementary material for: The COVID-19 Pandemic and Patient Safety Culture: A Cross-Sectional Study among Community Pharmacies in Jordan
Source: Healthcare (Basel). 2022 Jul 30;10(8):1434. doi: 10.3390/healthcare10081434 (PMC9408387; doi:10.3390/healthcare10081434)
Supplement: Supplementary file 1 [file healthcare-10-01434-s001.zip › Table S1.pdf]

Table S1. Patient Safety Culture Composites and Definitions

Composite	Definition: The extent to which…
Communication About Mistakes	Staff discuss mistakes that happen and talk about ways to prevent mistakes
Communication About Prescriptions Across Shifts	Information about prescriptions is communicated well across shifts, and there are clear expectations and procedures for doing so
Communication Openness	Staff freely speak up about patient safety concerns and feel comfortable asking questions; staff suggestions are valued
Organizational Learning—Continuous Improvement	The pharmacy tries to figure out what problems in the work process lead to mistakes and makes changes to keep mistakes from happening again
Overall Perceptions of Patient Safety	There is a strong focus and emphasis on patient safety, and the pharmacy is good at preventing mistakes
Patient Counseling	Patients are encouraged to talk to the pharmacist; pharmacists spend enough time talking to patients and tell them important information about new prescriptions
Physical Space and Environment	The pharmacy is well organized and free of clutter; the pharmacy layout supports good workflow
Response to Mistakes	The pharmacy examines why mistakes happen, helps staff learn from mistakes, and treats staff fairly when they make mistakes
Staff Training and Skills	Staff get the training they need, new staff receive orientation, and staff have the skills they need to do their jobs well
Staffing, Work Pressure, and Pace	There are enough staff to handle the workload, staff do not feel rushed, staff can take breaks, and work can be completed accurately despite distractions
Teamwork	The staff treat each other with respect, work together as an effective team, and understand their roles and responsibilities

Source: The Community Pharmacy Survey On Patient Safety Culture (PSOPSC) users' guide [31].
