# Supplementary material for: The COVID-19 Pandemic and Patient Safety Culture: A Cross-Sectional Study among Community Pharmacies in Jordan
Source: Healthcare (Basel). 2022 Jul 30;10(8):1434. doi: 10.3390/healthcare10081434 (PMC9408387; doi:10.3390/healthcare10081434)
Supplement: Supplementary file 1 [file healthcare-10-01434-s001.zip › Table S2.pdf]

Table S2. PRR on patient safety culture items and dimensions according to pharmacy ownership type (N=378)

SECTION A: Working in This Pharmacy	Independent Pharmacy N (%)	Chain Pharmacy N (%)	*P* value
**1. Physical Space and Environment**			
A1 This pharmacy is well organized	257 (88.0)	76 (88.4)	0.928 [b]
A5 This pharmacy is free of clutter	221 (75.7)	64 (74.4)	0.811[b]
A7 The physical layout of this pharmacy supports good workflow	241 (82.5)	67 (77.9)	0.332 [b]
**Dimension positivity**	719 (82.1)	207 (80.2)	0.896 [a]
**2. Teamwork:**			
A2 Staff treat each other with respect	274 (93.8)	81 (94.2)	0.905 [a]
A4 Staff in this pharmacy clearly understand their roles and responsibilities	258 (88.4)	77 (89.5)	0.762 [a]
A9 Staff work together as an effective team	261 (89.4)	71 (82.6)	0.089 [a]
**Dimension positivity**	793 (90.5)	229 (88.8)	**0.037** [a]
**3. Staff Training and Skills:**			
A3 Technicians in this pharmacy receive the training they need to do their jobs	232 (79.5)	69 (80.2)	0.874 [b]
A6 Staff in this pharmacy have the skills they need to do their jobs well	257 (88.0)	66 (76.7)	**0.009** [a]
A8 Staff who are new to this pharmacy receive adequate orientation	255 (87.3)	65 (75.6)	**0.008** [a]
A10 Staff get enough training from this pharmacy	241 (82.5)	66 (76.7)	0.227 [b]
**Dimension positivity**	985 (82.4)	266 (77.3)	0.325 [a]
**SECTION B: Communication and Work Pace**	**Independent Pharmacy N (%)**	**Chain Pharmacy N (%)**	***P* value**
**4. Communication Openness:**			
B1 Staff ideas and suggestions are valued in this pharmacy	232 (79.5)	57 (66.3)	**0.011** [b]
B5 Staff feel comfortable asking questions when they are unsure about something	242 (82.9)	67 (77.9)	0.294 [b]
B10 It is easy for staff to speak up to their supervisor/manager about patient safety concerns in this pharmacy	229 (78.4)	66 (76.7)	0.741 [b]
**Dimension positivity**	703 (80.3)	190 (73.6)	0.194 [a]
**5. Patient Counseling:**			
B2 We encourage patients to talk to pharmacists about their medications	256 (87.7)	73 (84.9)	0.499 [a]
B7 Our pharmacists spend enough time talking to patients about how to use their medications	242 (82.9)	72 (83.7)	0.854 [b]
B11 Our pharmacists tell patients important information about their new prescriptions	249 (85.3)	74 (86.0)	0.866 [a]
**Dimension positivity**	747 (85.3)	219 (84.9)	0.126 [a]
**6. Staffing, Work Pressure, and Pace**			
B3 Staff take adequate breaks during their shifts	193 (66.1)	33 (38.4)	**0.000** [b]
B9 We feel rushed when processing prescriptions (R)	74 (25.3)	25 (29.1)	0.490 [b]
B12 We have enough staff to handle the workload	231 (79.1)	59 (68.6)	**0.043** [b]
B16 Interruptions/distractions in this pharmacy (from phone calls, faxes, customers, etc.) make it difficult for staff to work accurately (R)	71 (24.3)	18 (20.9)	0.516 [b]
Dimension positivity	569 (42.9)	135 (39.5)	**0.018** [a]
**7. Communication About Prescriptions Across Shifts**			
B4 We have clear expectations about exchanging important prescription information across shifts	215 (73.6)	57 (66.3)	0.182 [b]
B6 We have standard procedures for communicating prescription information across shifts	196 (67.1)	59 (68.6)	0.797 [b]
B14 The status of problematic prescriptions is well communicated across shifts	228 (78.1)	66 (76.7)	0.793 [b]
Dimension positivity	639 (72.9)	182 (70.5)	0.124 [a]

Table S2. *cont.*

SECTION B: Communication and Work Pace	Independent Pharmacy N (%)	Chain Pharmacy N (%)	*P* value
**8. Communication About Mistakes:**			
B8 Staff in this pharmacy discuss mistakes	241 (82.5)	67 (77.9)	0.332 [b]
B13 When patient safety issues occur in this pharmacy, staff discuss them	225 (77.1)	67 (77.9)	0.868 [b]
B15 In this pharmacy, we talk about ways to prevent mistakes from happening again	239 (81.8)	62 (72.1)	**0.048** [b]
Dimension positivity	705 (80.5)	196 (76.0)	0.523 [a]
**SECTION C: Patient Safety and Response to Mistakes**	**Independent Pharmacy N (%)**	**Chain Pharmacy N (%)**	**P value**
**9. Response to Mistakes:**			
C1 Staff are treated fairly when they make mistakes	223 (76.4)	49 (57.0)	**0.000** [b]
C4 This pharmacy helps staff learn from their mistakes rather than punishing them	242 (82.9)	56 (65.1)	**0.000** [b]
C7 We look at staff actions and the way we do things to understand why mistakes happen in this pharmacy	240 (82.2)	66 (76.7)	0.258 [b]
C8 Staff feel like their mistakes are held against them (R)	184 (63.0)	36 (41.9)	**0.000** [b]
Dimension positivity	889 (76.0)	207 (61.2)	**0.001** [a]
**10. Organizational Learning—Continuous Improvement**			
C2 When a mistake happens, we try to figure out what problems in the work process led to the mistake	255 (87.3)	60 (69.8)	**0.000** [b]
C5 When the same mistake keeps happening, we change the way we do things	248 (84.9)	62 (72.1)	**0.006** [b]
C10 Mistakes have led to positive changes in this pharmacy	223 (76.4)	55 (64.0)	**0.022** [b]
Dimension positivity	726 (82.9)	177 (68.6)	**0.001**[a]
**11. Overall Perceptions of Patient Safety:**			
C3 This pharmacy places more emphasis on sales than on patient safety (R)	204 (69.9)	49 (57.0)	**0.026** [b]
C6 This pharmacy is good at preventing mistakes	252 (86.3)	66 (76.7)	**0.033** [b]
C9 The way we do things in this pharmacy reflects a strong focus on patient safety	256 (87.7)	63 (73.3)	**0.001** [b]
Dimension positivity	712 (81.3)	178 (69.0)	**0.006** [a]

(R): Negatively worded items were reversed coded; P-values of [a] Fisher's exact test and [b] Pearson chi-square test (numbers in bold are significant numbers generated from the statistical tests).
